# A systematic review on the cultural factors associated with stigma during pandemics

**DOI:** 10.1007/s12144-023-04509-0

**Published:** 2023-03-15

**Authors:** May Kyi Zay Hta, Rachel Sing-Kiat Ting, Pei Hwa Goh, Qian Hui Gan, Liz Jones

**Affiliations:** Department of Psychology, Jeffrey Cheah School of Medicine and Health Sciences, Monash University Malaysia, Jalan Lagoon Selatan, Selangor, Malaysia

**Keywords:** Pandemic stigma, Culture, Systematic review, Evolutionary psychology

## Abstract

Both public stigma and perceived self-stigma are prevalent during pandemics threatening a divide among the global community. This systematic review examined the cultural factors associated with viral respiratory-related pandemic stigma. Following PRISMA guidelines, the keywords, “culture, stigma, and pandemic” were searched across relevant databases for empirical papers between January 2000 to March 2022. Quality assessment and coding were adopted in the screening process. Thirty-one articles were included in the final analysis. Themes revealed that collectivistic values, cultural identities, and non-western regions were associated with public (others) stigma; mismatch of cultural values, minority groups, and North America, Asia, Oceania, and African regions were associated with higher perceived and self-stigma. We further mapped the themes into a proposed systemic cultural stigma model to integrate the dynamic intersection of cultural values, identity, and ecology. The cultural factors and their influence on stigma were then explained by drawing on two evolutionary theories: Cultural rationality theory and scapegoating theory. Lastly, we proposed culturally sensitive and responsive practices for stigma management at the community level, especially in non-Western regions during the pandemic recovery phase.

Throughout history, infectious diseases have emerged and spread across regions or populations as epidemics. “Epidemics occurring over a very wide area, crossing international boundaries and usually affecting a large number of people” are known as pandemics (Porta, 2014, p.209). Pandemics affect societies through physical illness and mortality, as well as a range of disruptions, including economic, social, and political consequences (Qiu et al., 2017). In particular, pandemics related to viral respiratory infections (VRIs), such as influenza (e.g., H1N1) or coronaviruses (e.g., severe acute respiratory syndrome [SARS], COVID-19), tend to trigger higher anxiety among the general population due to its life-threatening and highly contagious nature (Saeed et al., 2020). In addition, given VRIs’ ease of transmission through air and water droplets (Ong et al., 2020), public health control measures for VRI-induced pandemics generally require mass lockdown of public spaces and quarantine of high-risk groups. Such measures further reinforce public fear and isolation (Ren et al., 2020), which may leave individuals or groups associated with VRIs (e.g., countries of origin, highly infected regions, frontliners, patients, caretakers) especially vulnerable to stigmatization.

From the perspective of cultural psychology, scholars have proposed that culture plays an important role in stigmatizing attitudes and experiences, due to its underlying moral cognition (Yang et al., 2007). While there have been some commentaries highlighting the effect of culture on stigmatization during pandemics (e.g., Biswas et al., 2021; Bruns et al., 2020), there is still a gap in the synthesis of evidence-based studies to understand the cultural determinants of stigma during pandemics. Our study aimed to bridge this gap by conducting a systematic review of the published empirical articles in the past 21 years, to understand the role of culture on the perception of stigma towards others and self during VRIs pandemic. We aimed to advocate for culturally responsive and sensitive community services during the post-pandemic era, especially among the marginalized minorities.

## Stigma as social constructs

Stigmatization is defined as the differentiation and labeling of a collective group of individuals based on their characteristics, attributes, qualities, or status (Goffman, 1968). This usually leads stigmatized individuals or groups to experience social exclusion, discrimination, humiliation, and marginalization from the mainstream (e.g., Saeed et al., 2020). At the broadest level, stigma can be categorized into two distinct constructs, namely, public stigma and self-stigma (Corrigan & Watson, 2002). From a social psychological perspective, we adopted this broad definition of stigma in our systematic review, where public stigma refers to an individual expressing stigmatizing attitudes and behaviors towards others, and self-stigma is the perception of being stigmatized.

### Public stigma

Public stigma is defined as the negative beliefs and attitudes one holds that lead to fear, avoidance, rejection, or discrimination towards individuals with the disadvantaged condition (Corrigan & Penn, 1999; Parcesepe & Cabassa, 2013). In the context of the pandemic, the publicly stigmatized populations may include infected persons or those perceived to be at a higher risk of being infected, such as healthcare workers, family members, or people of a particular nationality or ethnicity, through associative stigma (Bhanot et al., 2021; Duan et al., 2020; Goffman, 1968; Hoppe, 2018). Public stigma, especially in the context of infectious diseases, can be explained through the evolutionary perspective of “othering,” with its function to protect oneself and one’s in-groups (Kurzban & Leary, 2001). Stigmatizing others is viewed as an act of adapting to increase the chances of both individual and in-group survival from the “perceived threat”—infected persons, or individuals or groups perceived to have associated attributes with the disease. To the individual, the phenomenon of separating between “them” and “us” allows the idea of distancing from perceived threat; the label “them” temporarily induces feelings of security and reduces fear and anxiety (Gilmore & Somerville, 1994; Saeed et al., 2020). This is especially prominent as epidemics and pandemics are characterized by and exacerbated by fear of the disease and uncertainty (Ren et al., 2020; Saeed et al., 2020). Hence, pandemics and virus outbreaks would be expected to be accompanied by social stigma toward others.

### Self-stigma

In this study, self-stigma refers to the awareness and perceptions of negative attitudes, prejudice, or discrimination others hold towards them (Corrigan & Rao, 2012; Link, 1987). The self-stigma model proposed by Corrigan and Rao (2012) argues that self-stigma may extend beyond initial awareness and perception of receiving stigma, to individuals internalizing those beliefs, leading to lower self-esteem. Sullivan et al. (2014) explained that scapegoating may occur in response to uncontrollable situations, usually towards the minority out-group, with the displacement of anger toward tangible and controllable targets in response to the uncontrollable situation. This is obvious during the pandemic, when the infected or “associated” people were blamed and discriminated against (e.g., Yang et al., 2021). As such, self-stigma usually results from the public projection of fear during the pandemic, where the stigmatized group (either infected or associated) becomes the scapegoat and the out-group. For example, during the COVID-19 pandemic, some of the self-stigma experiences ranged from feeling ashamed, being blamed, and being rejected, isolated, and abandoned by close ones, as well as the larger community (Duan et al., 2020; Nursalam et al., 2020; Yuan et al., 2021). Effects of self-stigma can also be long-term, extending beyond surviving the disease and even the pandemic itself. For example, SARS survivors struggled against stigma from family and co-workers (Lee et al., 2005), and those who were COVID-19 positive or quarantined reported self-stigma (e.g., feeling abandoned) even after recovery (Lohiniva et al., 2021; Nursalam et al., 2020). Hence, it is important to study the nature and mechanism behind self-stigma during both the pandemic and the recovery phase.

## Culture and stigma

On the socio-political level, previous research has identified several determinants that are associated with public stigma and self-stigma, including sociodemographic correlates such as education, health literacy, socio-economic status, gender, age, race/ethnicity, exposure to the virus, occupation as a healthcare worker, or the media (see, e.g., Yuan et al., 2021). We argue that culture is an additional factor that warrants a systematic study due to how the mechanism of culture interplays with pandemic stigma. While Link et al. (2004) viewed stigma as a universal phenomenon across different cultures and societies, Yang et al. (2007) highlighted that the experience and the endorsement of stigmatization could be very different and distinct, as stigmatizing actions, reasons, and outcomes differ based on culture. Moreover, some ethnic/racial identities or nationalities that are perceived to have a higher likelihood of being the carrier of virus or are believed to be the regional origin of the disease are highly stigmatized. (e.g., Bhanot et al., 2021; Hoppe, 2018). For example, literature reported that pandemics such as SARS and COVID-19 have been mainly associated with East Asians or Chinese, especially through media portrayal such as the news (e.g., He et al., 2020; Peprah & Gyasi, 2021).

On an individual level, there is some evidence that a person’s socio-cultural traits and norms may affect their perceptions and coping with a disease or illness. For example, Ji et al. (2020) reported how mainland Chinese had a more optimistic outlook towards the pandemic and the construct of suffering compared to the Euro-Canadians, potentially explained through dialectical thinking, whereby happiness and suffering co-exist in the Chinese population. Guan et al. (2020) also further explained how cultural constructs such as cultural orientation (e.g., collectivism/individualism—Hofstede, 1980; Triandis, 1995), cultural norms (e.g., tight/loose society—Gelfand et al., 2011), cultural-prone thinking styles (e.g., holistic/analytic—Nisbett et al., 2001) and self-construal (independent/ interdependent—Markus & Kitayama, 1991) impacted coping during the COVID-19 pandemic. Together, these different studies and approaches point to the close relationship between culture and perception of the pandemic, from the individual level to the national level, which warrants further studies from the cultural perspective (e.g., Yu et al., 2021).

## Research aims

In this current review, we defined “culture” as a group of people sharing the same membership, such as ethnicity, race, language, and religion, as well as having shared beliefs, values, social norms, ideologies, traditions, or customs (Ferraro, 1998; Hofstede, 1991; Matsumoto et al., 1996). To date, a few published commentaries have attempted to describe the role of culture on public stigma during the COVID-19 pandemic, such as discriminating against or blaming a specific cultural group (e.g., Biswas et al., 2021; Bruns et al., 2020). While certain cultural groups are more vulnerable to social rejection during a pandemic, there is a gap about what are the prominent cultural determinants of the exhibition of stigma towards the “culturally other”. Moreover, as both culture and pandemic stigma are complex and multifaceted constructs, measures of these constructs are not consistent in the existing literature. A systematic review is ideal for synthesizing findings through an operationalized definition of these constructs to summarize how culture may influence stigma, with culture as the major theme or independent variable, and pandemic stigma (both self-stigma and public stigma) as the outcome or dependent variable. This type of review is needed to prevent or mitigate pandemic stigma in the future, by designing culturally responsive health campaigns and policies. Therefore, we conducted a systematic review that aimed to examine cultural factors associated with public stigma and self-stigma during VRI pandemics.

## Method

### Search strategy

We conducted a rigorous systematic review following PRISMA guidelines (Moher et al., 2009) in the following electronic databases: OVID (including APA PsycArticles Full Text, APA PsycINFO), Scopus, and all databases in Ebscohost (including CINAHL), with criteria set to include peer-reviewed full-text journals in English, from January 2000 to the first week of March 2022, with human participants. The search terms for the three keywords, 1) culture, 2) stigma, and 3) pandemic, were brainstormed and discussed between the authors based on the thesaurus and online searches, as shown in Table 1. We adopted wider definitions of all these three key terms to be exhaustive in our initial search, especially the types of pandemics being researched.

**Table 1:** Search Terms for Main Keywords and Databases Used

	Culture	Stigma	Pandemic
Search terms for main keywords	Ancestr* Belief* Cultur* Countr* Cross-cultur* Cross-nation* Cross-geographic* Custom* Ethnic* Indigen* Language* Multi-cultur* Nation* Rac* Religio* Ritual* Social norm Social norms societ* Tradition*	Stigma* Discriminat* stereotyp* Prejudice* attitude* Perception* Shame Blame Fear	Airborne Coronavirus* Covid* Epidemic Flu H1N1 H5N1 Influenza MERS Pandemic SARS SARS-Cov-2 2019-nCoV
Databases	Ovid (including APA PsycArticles Full Text, APA PsycINFO) (N = 38,966) Ebscohost (N = 13,342) Scopus (N = 18,429)		

### Study selection and eligibility criteria

Only studies fulfilling the following criteria were included:

- (1) Full-text articles in the English language.
- (2) Publications in peer-reviewed journals online from January 2000 to the third week of March 2022.
- (3) Studies with human participants (i.e., content analysis of social media or news were excluded).
- (4) Empirical studies—quantitative, qualitative, or mixed-methods (authors’ views, commentaries, theoretical papers, systematic reviews or meta-analyses, book chapters, conference proceedings, dissertations, and theses were excluded).
- (5) Studies examining culture as an independent variable and stigma as a dependent variable in the context of a pandemic.
- (6) Studies of viral respiratory infectious disease at pandemic or epidemic level only (i.e., coronavirus or influenza) (non-respiratory pandemic/epidemic such as Human Immunodeficiency Virus (HIV) or Ebola infections were excluded).

**Fig. 1:**
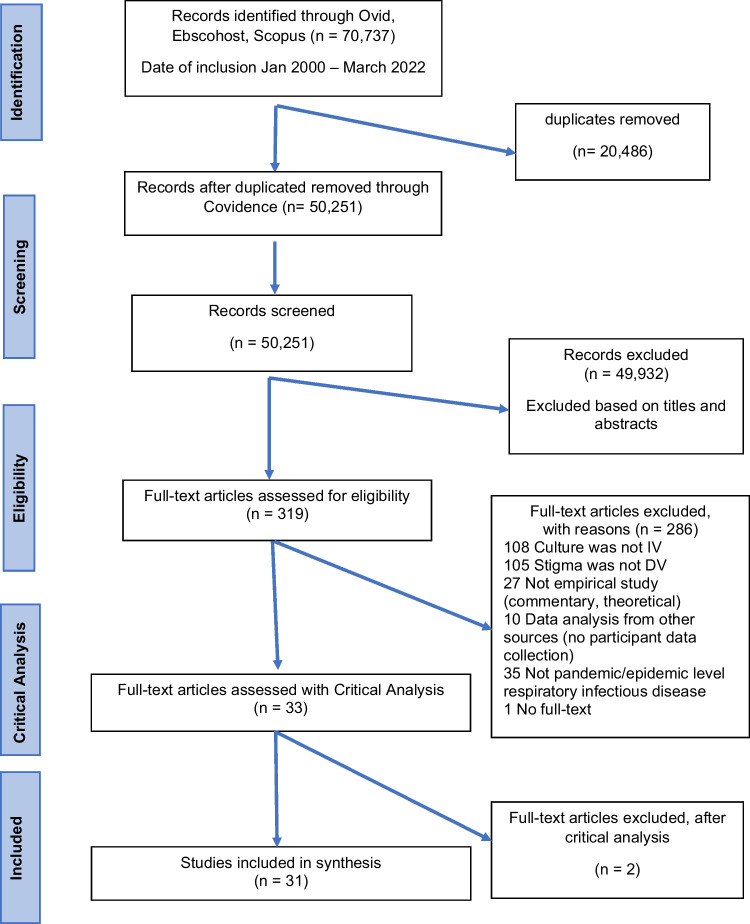
Preferred Reporting Items for Systematic Reviews and Meta-Analyses (PRISMA) Flow Chart of Systematic Review

The screening process was reported in detail as per Preferred Reporting Items for Systematic Reviews and Meta-Analyses (PRISMA) reporting guidelines (Moher et al., 2009), as shown in Fig. 1. The initial literature search yielded 70,737 research articles across the 3 databases: Ovid (N = 38,966), Ebscohost (N = 13,342) and Scopus (N = 18,429). The citations and abstracts were first imported into Endnote X9, which were further imported into the online Covidence platform to remove duplicates. After removing duplicates in Covidence, 50,251 research articles were left for further screening. The research team (consisting of the first and fourth authors and six research assistants) screened each title and abstract for relevance to the systematic review based on the inclusion and exclusion criteria, and excluded 49,932 articles in the preliminary screening (most of them were non-VRI diseases such as HIV). The team then reviewed the remaining 319 articles in full text where 286 articles were further excluded due to not fulfilling the screening criteria above.

### Critical appraisal of the selected articles

The remaining 33 articles were further reviewed independently by the first and third authors, using Hawker’s critical appraisal ratings (Hawker et al., 2002), and rated as very poor, poor, fair, or good (score 1 to 4) for each of the following nine sections: abstract and title, introduction and aims, method and data, sampling, data analysis, ethics and bias, findings/results, transferability/generalizability, and implications and usefulness (see Table 2). The ratings from the initial review were averaged, and the discrepancy was reconciled by the second author. Two studies were excluded due to poor quality in their methodology (less than the cut-off score of 21, max = 36). Finally, 31 studies were included for the final synthesis in this study.

**Table 2:** Hawker’s Score for Critical Appraisal of Articles (Averaged Scores from 2 Independent Raters with Likert Scale from 1–4)

Author (year)	Abstract & Title	Introduction & Aims	Method & Data	Sampling	Data Analysis	Ethics & Bias	Findings/Results	Transfer-ability/Generali-sability	Implications & Usefulness	Total score (out of 36)	Average score (out of 4)
Ahuja et al. (2020)	3.5	3	3.5	2.5	3.5	3.5	3.5	3	3.5	29.5	3.28
Dollman & Kogan (2021)	3	3	3.5	2.5	3.5	1	3	3.5	3.5	26.5	2.94
Dye et al. (2020)	3.5	2.5	3.5	3.5	4	4	3.5	3.5	4	32	3.56
Eichelberger (2007)	3	3.5	3	2	2.5	2	3	2.5	3.5	25	2.78
Haddad et al. (2021)	3.5	3	3.5	3	3.5	3.5	3	3	2.5	28.5	3.17
Heinnen et al. (2021)	3	2	3	2.5	3.5	3.5	3	3	3	26.5	2.94
Inman et al. (2021)	3	2.5	3	2.5	3.5	3	3.5	3.5	3	27.5	3.06
Islam et al. (2021)	2.5	4	3.5	2.5	4	3.5	3.5	3.5	4	31	3.44
Ji and Chen (2022)	3.5	4	2.5	3	3.5	4	3	3	3	29.5	3.28
Jiang et al. (2021)	3	2.5	3.5	3.5	3	3.5	3	3.5	3	28.5	3.17
Jesuthasan et al. (2021)	3.5	3.5	4	3.5	3.5	3.5	3.5	3	3	31	3.44
Kim et al. (2022)	3	3	3	2.5	3	4	3	3	3	27.5	3.06
Le et al. (2022)	4	3	3.5	2	4	1.5	3.5	3.5	3.5	28.5	3.17
Li and Chen (2021)	3	4	3	4	3	3.5	3	3	3	29.5	3.28
Ma and Zhan (2020)	3	4	3	3.5	2	1	4	3	4	27.5	3.06
Miconi et al. (2021a)	4	3.5	3.5	3.5	3.5	3.5	3.5	3	3.5	31.5	3.50
Miconi et al. (2021b)	4	3.5	3.5	3	3.5	3	3	3.5	3.5	30.5	3.39
Molock and Parchem (2021)	3.5	3.5	3.5	2	3	3	3	2.5	2.5	26.5	2.94
Oh and Min (2022)	3	3.5	4	3.5	3.5	3.5	3.5	3	3	30.5	3.39
Pan et al. (2021)	4	3.5	3.5	2.5	4	3	3.5	3.5	4	31.5	3.50
Park et al. (2022)	3.5	3	4	3	4	3.5	3.5	4	3	31.5	3.50
Schmidt et al. (2020)	3.5	3	4	3	3.5	4	3.5	3	4	31.5	3.50
Shah et al. (2021)	3	4	3.5	2.5	3.5	3.5	4	3	3	30	3.33
Shang et al. (2021)	3.5	3.5	3.5	3.5	3.5	4	4	3.5	3.5	32.5	3.61
Strassle et al. (2022)	4	2.5	3.5	3.5	4	2	4	3.5	3	30	3.33
Tan and Umamaheswar (2021)	3	3.5	3	3.5	3.5	3.5	3.5	4	3	30.5	3.39
Vandrevala et al. (2022)	4	3	3	3	4	3	3.5	3.5	4	31	3.44
Wang et al. (2021)	2.5	3	3.5	2.5	3	3.5	3	3.5	2.5	27	3.0
Weiden et al. (2021)	3.5	3.5	3.5	2	3	4	3	3.5	3	29	3.22
Wong & Sam (2010)	3	2	3.5	3	3.5	3	3	3	3.5	27.5	3.06
Zong et al. (2021)	3.5	4	4	3	4	3.5	4	3.5	3.5	33	3.67

### Qualitative coding of the findings

The above 31 articles were coded using a bottom-up inductive method through thematic analysis (Braun & Clarke, 2006) by the first and second authors. Next, the themes were discussed and agreed upon by all authors following a consensual qualitative research (CQR) approach, using bottom-up coding (Hill et al., 2005) to minimize individual biases in interpretation, based on the research question.

## Results

### Study characteristics

As seen in Table 3, the majority of the studies were conducted in North America (n = 19), with 16 from the United States of America (USA), two from Canada, and one from the USA and Canada. Others came from diverse regions in Europe (n = 3), Asia (n = 6), Australia (n = 1), and South Africa (n = 1). One study included 173 countries worldwide, with regional comparisons. Most studies were conducted during the COVID-19 pandemic (n = 29), with the rest conducted during SARS (n = 1) or H1N1 (n = 1). Studies relied on quantitative (n = 17), qualitative (n = 10), and mixed (n = 4) methods. Most studies examined self-stigma (n = 26), while only seven examined public stigma (n = 7) as the outcome variable/s. Other details, including participants’ characteristics and measurements, are presented in Table 3.

**Table 3:** Characteristics of the 31 Selected Articles for Systematic Review

	Authors (year)	Location	Participant Demographics (N)	Type of pandemic	Type of Study	Measurement of Culture	Measurement of Stigma (Public Stigma [PS] vs. Self-Stigma [SS])
1	Ahuja et al. (2020)	India	The general adult population of non-Islamic Indians (600) Mean age: 38.76 years (18–83 years) Gender: 231 males, 366 females, and 3 others Religion: Hindus (4.8%), Sikhs (2.8%), Christians, and others (5.6%) from 26 states/union territories (UTs), representing nearly all parts of India	COVID-19	Quantitative	Collectivism scale (Kim et al., 2016)	PS: Specific xenophobia scale (adapted by authors)—pandemic stigma towards Muslims during COVID-19 in the Indian context
2	Dollman & Kogan (2021)	Germany	Young adults born between 1994 and 1996 and aged between 24–26 (3517) Country of origin: German (1,905), Former Soviet Union countries (197), Central and Eastern European countries (442), Other European countries (273), North and South America (61), East and Southeast Asia (80), Middle East (including Turkey) and Africa (507)	COVID-19	Quantitative	Self-reported country of origin and ethinicity	SS: Self-reported discrimination through questions on COVID-19–associated discrimination (CAD) (Soin´e et al., 2021) “Since the beginning of the Corona pandemic, do you feel increasingly discriminated against or treated unfairly due to your ethnic background?”
3	Dye et al. (2020)	Multiple countries (174) (six regions across the world)	General adult population from 173 countries (7411), including HCW (837)	COVID-19	Mixed method	Self-reported country of residence	PS: Two PS questions (adapted from STRIVE (Stangl et al., 2018) SS: Close-ended question on whether one has experienced COVID-19 related harassment/ bullying/ discrimination with open-ended text for elaboration
4	Eichelberger (2007)	United States	People in Chinatown (majority Chinese) of US No demographic information of participants reported	SARS	Qualitative	Self-reported ethnicity (in some quotes)	SS: Through participant observations for 6 weeks and semi-structured, narrative interviews (no sample provided)
5	Haddad et al. (2021)	Lebanon	The general adult Lebanese population (405) Mean age = 28.38 years Gender: Male (20.2%), Female (79.8%) Education: School (10.2%), University (89.6%) Religion: Christian (17.5%), Muslim (62.5%), Druze (10.4%), Atheist (0.2%), Refuse to answer (8.6%), Other (0.5%)	COVID-19	Quantitative	Self-reported religion	PS: Stigma Discrimination Scale (5-point Likert scale) – measuring discriminatory attitudes towards people with COVID-19. Adapted from (Green, 1995; Genberg et al., 2008; See et al., 2011) E.g., “People with current COVID-19 are dangerous to the society”, “People with current COVID-19 should not have the same freedoms as other people.” SS: Self-stigma scale adapted from COVID-19-related stigma scale (Do Duy et al., 2020) and HIV Berger scale (Berger et al., 2001) E.g., “I feel guilty because of being isolated” & “I have been hurt by how people reacted to learning I have COVID-19”
6	Hennein et al. (2021)	United States	Healthcare workers employed at academic hospitals located in 12 states with high rates of COVID-19 in the US during pandemic (997) Age: Mean 38.22 (SD 11.77) years Gender & Ethnicity: Females underrepresented in medicine (URM) (12.2%), URM males (4.0%), Asian females (10.1%), Asian males (4.7%), non-Hispanic White females (49.1%), non-Hispanic White males (19.8%)	COVID-19	Mixed method	Self-reported race/ethnicity	SS: Quantitative: General Ethnic Discrimination Scale (Landrine et al, 2006) Qualitative: Open-ended questions asking about experiences of racial and ethnic discrimination
7	Inman et al. (2021)	United States	Undergraduate and graduate university students in a large public university in US during pandemic (1261) Average age: 22.4 years Gender: Female (69.5%) Race: non-Hispanic White (60.2%), Asian (39.8%)	COVID-19	Quantitative	Self-reported race identity	SS: (1) Everyday Discrimination Scale (Williams et al., 1997) to assess the experienced discrimination in past month with seven open-ended recall questions to remember the events (2) Questions to capture the frequency of verbal harassment on E.g., “you were treated with less respect than others”
8	Islam et al. (2021)	India	General adult population 40 localities across the Kanpur Nagar district of Uttar Pradesh in India (2138) Age: Mean 38.66 years (SD 12.22) Gender: Male (53%) Religion: Hindu (79%) Caste background: Low-caste (63%)	COVID-19	Quantitative Baseline survey and educational intervention	Self-reported religion	PS: Stigma index (5-point Likert scale): questions about stigmatization of COVID-19 patients and their family members and biased belief or prejudice against religious/vulnerable groups
9	Ji and Chen (2022)	United States	Chinese International students residing in US during pandemic (10) Age: 20–30 Gender: Female (5), Male (5) Ethnicity: Chinese Religion: Atheist (7), Unspecified (2), Buddhism (1)	COVID-19	Qualitative	Self-reported ethnicity	SS: Semi-structured interviews on own and friends’ experience of being stigmatized
10	Jiang et al. (2021)	China	General adult population in 31 provinces in China during pandemic (5039) Age: average 33.0 years (SD = 12.5) Gender: Female (58.5%) Ethnicity: Han (84%), Minorities (16%)	COVID-19	Quantitative	Self-reported ethnicity	PS: PS against Wuhan residents were measured by questionnaire adapted from previous studies (Chowdhury et al, 2015; Datiko et al., 2020) e.g. “It is their problem and I don’t want to get COVID-19 by trying to help them,” “I am afraid of them and avoid them because they may infect me,” (classified as stigmatized)
11	Jesuthasan et al. (2021)	United States	Adult public healthcare workers with ethnic minority background (13) Age: Mean 42.7 years (SD = 10.3) Gender: Female (11) Ethnicity: Eastern European (1), Indian (2), Mixed White and Black Caribbean (1), Caribbean (1), Mixed White and Asian (1), African (2), Arab (1), Bangladeshi (1), Asian Other (2) and Other- Mixed North African and Eastern European (1)	COVID-19	Qualitative	Self-reported ethnicity	SS: Focus group discussion with study interview guide to access experiences of ethnic minority healthcare workers
12	Kim et al. (2022)	United States (Florida)	General adult population in Florida, US during the pandemic (221) Qualitative: Asians with racial discrimination experience (39) Age: 18–24 (20.2%), 25–34 (25%), 35–44 (15.5%), 45–54 (17.9%), 55–64 (16.7%), 65 + (4.8%) Gender: male (32.7%) Ethnicity: Asian (163), Non-Asian (53)	COVID-19	Mixed method	Self-reported race, country of birth	SS: Quantitative: Experienced and anticipated discrimination (adapted from Council APPP, 2020) Qualitative: Open-ended field for elaboration for participants who reported discrimination “the same as” or “more than before the pandemic”
13	Le et al. (2022)	United States (Five southern cities)	General adult population in adult residents of five U.S. metropolitan statistical areas (MSA) in the South: Atlanta-GA, Austin-TX, Dallas-TX, Houston-TX, and New Orleans-LA during pandemic (1688) Race/Ethnicity: Hispanic (any race) (26.6%), 44.3% non-Hispanic White, 21.8% non-Hispanic Black, and 7.4% non-Hispanic Asian	COVID-19	Quantitative	Self-reported race/ethnicity	SS: Adapted Everyday Discrimination Scale (5 items) on daily slights and harassment (Sternthal et al., 2011) (2) Adapted Major Experiences of Discrimination Scale (8 items) (Williams et al., 1997)
14	Li and Chen (2021)	Australia	Chinese queer women (age 19–37 years), from originating from Mainland China, Hong Kong, Taiwan, Malaysia, Singapore, and Vietnam, residing in Australia for 7 months to 9 years during pandemic (31)	COVID-19	Qualitative	Self-identified as Chinese women	SS: Semi-structured and in-depth interviews and participant observation through WeChat (through audio and video chats) or Zoom, face-to-face to understand experiences of racial discrimination in the digital dating apps
15	Ma and Zhan (2020)	United States	Chinese students in the USA or who studied in the USA during pandemic (30) Age: 18—21 years Gender: females (16), males (14)	COVID-19	Qualitative	Self-reported ethnicity	SS: Through semi-structured and in-depth phone interviews (e.g., “what reactions [Chinese students] have received from Americans about wearing masks,” “how [Chinese students] cope with those reactions.”)
16	Miconi et al. (2021a)	Canada	General population Quebec residents (3273) Age: aged 18–39 years (49%) Gender: female (57%), male (43%) Ethnicity: White (49.07%), East Asian (7.61%), South Asian (2.93%), Black (21.14%), South-East Asian (3.64%), Arab (13.75%), Other (1.86%)	COVID-19	Quantitative	Self-reported ethnocultural groups	SS: Scale for COVID-19-related perceived discrimination across binary points (Yes/No) (adapted from Williams et al., 1997)
17	Miconi et al. (2021b)	Canada	General population Quebec, Canada residents (3273) Mean age = 42.74, SD = 16.95, range: 18–88; Gender: female (57%), male (43%) Ethnicity: White (49.07%), East Asian (7.61%), South Asian (2.93%), Black (21.14%), South-East Asian (3.64%), Arab (13.75%), Other (1.86%)	COVID-19	Quantitative	Self-reported ethnocultural groups	SS: Scale to measure context and reasons for perceived COVID-19 related discrimination across binary points (Yes/No) (adapted from Williams et al., 1997)
18	Molock and Parchem (2021)	United States	Ethnic minorities in the USA Mean age: 20.52, range (18–24) Gender: males (49%) and females (49%), Preferred not to disclose gender (1 participant) Ethnicity: Black (46%), Asian, American/Pacific Islander (21%), Latino/a (18%), Multiracial (7%), White (5%), West Indian (2%)	COVID-19	Quantitative	Self-reported ethnicity	SS: A set of close-ended (Yes/No) and open-ended questions consisting of 16-items created by the authors (e.g., personally experienced or witnessed acts of racial discrimination as a result of the COVID-19 pandemic)
19	Oh and Min (2022)	United States	Asian American journalists (20) Gender: male (8), female (12)	COVID-19	Qualitative	Self-reported racial and ethnic identification	SS: Online in-depth interviews (1) what Asian American identity means to them, (2) how their identities matter in the newsroom and in their practice of news, and (3) what it meant to their news work to be visibly marked as Asian American during the pandemic
20	Pan et al. (2021)	United States	General US residents adult population (above 18 years) (6707) Ethnicity: Non-Hispanic White (63.4%), Hispanic White (13.9%), Black (12.3%), Asian only foreign-born (3.6%), Asian only US-born (1.9%), Asian of mixed race (1.5%), non-Asian of mixed race (2.4%), Indigenous only (1%)	COVID-19	Quantitative	Self-reported racial affiliation Self-reported ethnicity Self-reported Foreign-born status	SS: coronavirus stigma experience questions created by the authors (yes/no/unsure) (1) Treated with less courtesy and respect; (2) received poorer service at restaurants or stores; (3) caused other people to be afraid of the participant; (4) received threats or harassment
21	Park et al. (2022)	United States	Asian Americans and Pacific Islanders (4971) Mean age: 45.2, range: 18–97 Females (64.1%) Ethnic Chinese (including persons from China, Hong Kong, and Taiwan; (33.9%), Korean (22.5%), and Vietnamese (19.3%) Foreign-born (63.4%)	COVID-19	Quantitative	Self-reported ethnicity	SS: Scale measuring discriminatory experiences during the pandemic through adapted Everyday Discrimination Scale (EDS) (Shariff-Marco et al., 2011; Williams et al., 1997)
22	Schmidt et al. (2020)	South Africa (Gauteng, KwaZulu-Natal and the Western Cape provinces)	Key informant interviewees (60) Age: above 18 years Gender: cisgender women (36), cisgender men (22), and transgender women (2) Focus group discussion (5), cisgender women (2) and cisgender men (3)	COVID-19	Qualitative	Self-reported ethnicity	PS: Key informant interviews and focus group with one of the aims being exploring stigmatization related to COVID-19 pandemic (Probe, e.g., “…would you be treated differently to those who were not infected? If so, in what way and how?”
23	Shah et al. (2021)	United States	College students (1249) from Georgia Southern University Age: Mean 22.6 (22.2–22.9) years Gender: Female (66.6%), Male (32%), Nonbinary (1.4%) Race: Black (17.4%), White (50.3%), Asian, multiple races, and others (32.3%)	COVID-19	Quantitative	Self-reported race	SS: Scale (5-point Likert) on experiencing acts of bias and hatred during the COVID-19 “If you have experienced acts of bias or hatred related to your race or skin color, how often has this happened since the onset of COVID-19?”
24	Shang et al. (2021)	Canada & United States	Healthcare workers from US (15) and Canada (15) (at least 1 year of full-time employment, who self-identified as Asian): nurses (16), attending physicians (5), physiotherapists (3), resident physicians (2), midwife (1), paramedic (1), pharmacist (1), physician assistant (1) Age: 25–29 years (53%) Gender: Female (60%), Male (37%), Non-binary (3%) Ethnicity: Chinese (27%), Filipino, Filipina (17%), Vietnamese (17%), Taiwanese (10%), Cambodian (7%), Korean (7%), Multiracial (7%), Japanese (3%), Malaysian (3%), Pakistani (3%)	COVID-19	Qualitative	Self-reported ethnicity	SS: Semi-structured interviews exploring experiences of discrimination focusing on microaggressions faced by Asian health care professionals in US and Canada e.g., “Tell me about your experiences as an Asian Canadian/American during the COVID-19 pandemic”
25	Strassle et al. (2022)	United States	US nationally representative adults (5500) Age: 18 years and older Asian (1000), Black/African American (1000), Latino (1000 – Spanish speaking (500), English speaking (500)), White (1000), American Indian/Alaska Native (500), Hawaiian/pacific Islander (500), Multiracial (500)	COVID-19	Quantitative	Self-reported ethnicity	SS: Scale assessing COVID-19 related discrimination, adapting everyday discrimination scale (William et al., 1997)
26	Tan and Umamaheswar (2021)	United States	Undergraduate & graduate students of highly diverse (socioeconomic & racial) university in northeast of US (36) Age: 18–24 (63.8%), 25–39 (25%), 40–59 (5.5%), 60 + (5.5%) Gender: Women (58.3%), Men (38.8%), Other (2.7%) Race/Ethnicity: Black (44.4%), Latinx (19.4%), White (36.11%)	COVID-19	Qualitative	Self-reported race/ethnicity	SS: semi-structured interview contained mostly open-ended questions related to three thematic areas: Risk perceptions; experiences with uncertainty; and experiences with shelter-in-place orders
27	Vandrevala et al. (2022)	United Kingdom	Ethnic minorities in the UK (57) Age: 18 and above Race/Ethnicity: Black – African/Caribbean (28), South Asian – Pakistani/Bangladeshi/Indian (29)	COVID-19	Qualitative	Self-reported ethnicity	SS: Semi-structured interviews (online video conferencing/telephone) – exploring perception of how COVID-19 pandemic has affected the individual and their community
28	Wang et al. (2021)	France	Chinese in France (student alumni, Chinese descendants in France) Online survey (255) Interview (21) Age: Median = 26 (30 years old or younger (2/3), older adults, skilled migrants who can also be parents of young children born in France, etc.)	COVID-19	Mixed method	Self-reported nationality and migratory status	SS: Questions designed by authors including assessing social environment and experiences of discrimination experiences during COVID-19 (e.g., discriminatory treatment during the pandemic—Yes/No/I don’t know) with open-ended text fields for elaboration; interviews for other participants from the epistemological approach
29	Weiden et al. (2021)	Israel	Ultra-Orthodox Jewish families in Israel (252) Age: Mean 32.85 (SD 10.63)years Gender: Female (67%)	COVID-19	Quantitative	Self-reported religiosity level as ultra-orthodox Jew	SS: The Racial and Ethnic Microaggressions Scale (REMS) (Nadal 2011) E.g., “Someone else was making it clear that he is afraid of getting infected by me because I am an ultra-Orthodox Jew”
30	Wong & Sam (2010)	Malaysia	Malaysian residents from Selangor state and the Federal Territory of Kuala Lumpur (1050), > 18 years Ethnicity: Malay (41.5%), Chinese (33.5%), Indians (25%) Religion: Muslim (41.9%), Buddhist (23.7%), Taoist (1.6%), Hindu (22.5%), Sikh (0.2%), Christian (7.4%), No religion (2.0%), Others (0.7%)	H1N1	Quantitative	Self-reported ethnicity	PS: Computer-assisted telephone interview on A(H1N1)-related stigma (adapted by the authors based on past literature)—e.g., afraid to be in contact with people who had just returned from overseas
31	Zong et al. (2021)	United States	Chinese American adolescents in US (213) Age: 10—18 years (Mean = 13.95 years, SD = 2.35) Gender: Girls (49%) Country of birth: United States (80%), Mainland China (16%), Hong Kong (1%), Taiwan (< 1%), other places (3%) Residence in US: Southern (76%), Northeastern (9%), Midwestern (6%), Western (6%), did not report residence (3%)	COVID-19	Quantitative	(1) Parents-reported Ethnicity (2) Ethnic identity – adapted Multidimensional Inventory of Black Identity (Sellers et al., 1997) (3) Bicultural identity integration Version 2 (Huynh et al., 2018) (4) Behavioural Acculturation measured by Cultural and Social Acculturation Scale (Chen & Lee, 1996)	SS: Direct discrimination assessed by (1) Online Victimization Scale (4 items) (Tynes et al., 2010)—e.g., “People have said mean or rude things about me because of my race or ethnic group online” and (2) Racial and Ethnic Microaggressions Scale (4 items) (see, 2011)—e.g., “Some people were unfriendly or unwelcoming toward me because of my Chinese background” Vicarious discrimination assessed by (1) Online vicarious discrimination (adapted from Tynes et al., 2010)—e.g., “People have said things that were untrue about people in my race or ethnic group online” and (2) Asian American Racism-Related Stress Inventory (Miller et al., 2012)—e.g., “People have said things that were untrue about Chinese people because of the COVID-19 outbreak”

### Cultural factors and pandemic-related public stigma

From the seven studies that examined public stigma (e.g., discriminatory attitudes), four cultural factors were identified: Race or Ethnicity, Religious Identity, Collectivistic Values, and Residence in Non-Western Countries (see Table 4).

**Table 4:** Cultural Factors Associated with Public Stigma (PS) during Pandemics caused by Respiratory Viruses

Themes	Extracted Findings	Authors (year)
1. Race/Ethnicity	In Malaysia, Malays, compared to Chinese and Indians, were seen to have higher fear towards people/situations associated with A(H1N1) (public stigma), such as being afraid of contacting those who arrived from overseas or afraid of eating outside	Wong and Sam (2011)
	In South Africa, some participants believed that being Blacks made them less vulnerable to COVID-19 and that it is a “white-mans' disease” or for wealthy people who travel overseas“…this virus is for wealthy people, who have money and ‘white’ people, not Africans or ‘blacks” (p.12)“Some people used to say that it can’t affect ‘black’ people. They thought it was for ‘white’ people. …it doesn’t affect ‘black’ people, we have strong genes and so on” (p.12)	Schmidt et al. (2020)
	In China, those who were of ethnic minorities were seen to exhibit higher public stigma (fear and avoidance) towards people from Wuhan and patients with COVID-19 compared to the Han majority	Jiang et al. (2021)
2. Religious Identification	In Lebanon, being a Muslim or a Druze was seen to be associated with higher public stigma (discriminatory attitude) towards those infected with COVID-19 compared to Christians	Haddad et al. (2021)
	In India, Hindus perceived Muslims to be responsible for the spread of COVID-19. However, the opposite was not true as the Muslims did not perceive Hindus to be responsible for the spread of the COVID-19	Islam et al. (2021)
3. Collectivism	Collectivism was positively correlated with specific xenophobia (public stigma) towards Muslims by non-Muslims in India during the COVID-19 pandemic	Ahuja et al. (2020)
4. Non-Western Countries	Asia, Africa, Latin America & Caribbean regions reported higher pandemic-related public stigma (overall negative perception including losing status or respect of those associated with COVID-19 such as infected or family members of the infected) compared to Europe, Northern America, or Oceania	Dye et al. (2020)

[Insert Table 4: Cultural Factors Associated with Public Stigma during Pandemics].

#### Race and ethnicity

As shown in Table 4, three studies found that being members of the majority race or ethnicity was associated with public stigma. In Wong and Sam’s study (2011), ethnicity was associated with the extent to which participants endorsed stigmatizing attitudes during the H1N1 pandemic. They found that among Malaysians, Malays endorsed the highest public stigma in the domain of fear (such as being afraid of those from overseas or eating outside) compared to Chinese and Indians. A qualitative study by Schmidt et al. (2020) also found that ethnic or racial identity was associated with public stigma towards other ethnicities during the COVID-19 pandemic, whereby some of the Blacks in South Africa believed COVID-19 to be a “White-mans’ disease” or a disease for those in China or Italy. They believed that the disease only affected “… upper-class people who travel a lot”, “it was for the White people” or that “it doesn’t affect Black people, we have strong genes…” (Schmidt et al., 2020, p.12). On the other hand, a study in China by Jiang et al. (2021) found that ethnic minorities expressed higher public stigma, such as fear and avoidance towards COVID-19 patients and people from Wuhan, compared to the Han majority. Overall, these studies demonstrated that racial and ethnic identities were associated with negative perceptions of others who were affiliated with the pandemic, especially those from other racial groups.

#### Religious identity

Two studies found different religious identities within a country were associated with differences in public stigma during the COVID-19. In Lebanon, those who identified as a Muslim or a Druze reported higher discriminatory attitudes towards those infected with COVID-19 compared to the Christians (Haddad et al., 2021). A study in India by Islam et al. (2021) found that while Hindus perceived Muslims to be responsible for the spread of the COVID-19, Muslims did not hold this perception towards the Hindus.

#### Collectivistic values

Findings from Ahuja et al. (2020) showed a positive relationship between collectivism and pandemic-related public stigma in India, where collectivism was defined as the extent to which family and society needs take importance over an individual’s needs (Kim et al., 2016). They found that the more the non-Muslim participants endorsed collectivist values, the more they expressed public stigma (blaming and attributing the spread of the COVID-19 pandemic) towards Muslims in India.

#### Non-Western countries

A global survey conducted by Dye et al. (2020) across 173 countries found differences in COVID-19 related public stigma across different geographical regions. In comparison to participants from Europe, Northern America, or Oceania, participants from Non-western countries such as Asia, Africa, Latin America, and Caribbean regions were more likely to believe that individuals in their community had a negative perception of those who were infected or associated with COVID-19. They were also more likely to believe that people lost status and respect in the community when they tested COVID-19 positive compared to participants in Europe, Northern America, or Oceania. However, Dye and colleagues (2020) did not explain why there were differences across the regions.

### Cultural factors and pandemic-related self-stigma

Out of 26 articles, we identified five cultural factors which had self-stigma during the pandemic (e.g., perceived stigma/discrimination, harassment) as the outcome variable: Minority by Race, such as the overseas Chinese, Minority by Religion, Minority in Spoken Language, Cultural Values that conflicted with the Dominant Society, and Countries in Northern America, Africa, Oceania, and Asian regions (see Table 5).

**Table 5:** Cultural Factors Associated with Self-Stigma (SS) during Pandemics caused by Respiratory Viruses

Themes	Extracted Findings	Authors (year)
1. Ethnic Minorities (especially Chinese) living in Western Countries	During the COVID-19 pandemic, significant differences in perceived self-stigma was reported in Canada across ethnicities, according to prevalence rate: East Asian > South Asian > South-East > Asian > Black > Other > Arab > White	Miconi et al. (2021a)
	During the COVID-19 pandemic, significant differences in the perceived self-stigma was found across ethnicities in Canada, according to the odds ratio: East Asian > South Asian > Southeast Asian > Other > Black > Arab > White The most reported reason for discrimination was “race/ethnicity,” according to odds ratio in the following order: East Asians > South-East Asian > South Asian > Black > White	Miconi et al. (2021b)
	During the COVID-19 pandemic, in the US, among People of Color [POC], differences were seen in reported frequency of witnessing or experiencing self-stigma across ethnicities in the following order of descriptive statistics (%): Asian > Black > ‘other’ > Latino/a	Molock and Parchem (2021)
	In the US, foreign-born Asian, US-born Asian, Black, and Hispanic Whites reported experiencing self-stigma significantly more than non-Hispanic Whites during the COVID-19 pandemic	Pan et al. (2021)
	In a global study of over 173 countries, Asian or Chinese participants reported feeling stigmatized/discriminated against because of their ethnicity during the COVID-19 pandemic “Seen a lot of racist graffiti around my city towards Asians. Also heard [racist slur] a few times.” (Participant from East Asia, residing in Northern America, p.10) “I am Asian…people treated me badly on the street and avoided me” (translated, participant from Southeastern Asia living in Southwestern Europe, p.10)	Dye et al. (2020)
	During the SARS pandemic, Chinatown resident in the US reported feeling stigmatized during SARS pandemic due to his ethnic identity of being Asian “I did feel more self-conscious that I was getting these looks because I was—I am Asian, Asian–American. That was hard for me.” (p.1290)	Eichelberger (2007)
	Chinese in France reported self-stigma due to being Asians, during the COVID-19 pandemic. Reasons specifically mentioned by the participants included–Asian physical appearance, China as pandemic origin and for a few, their accent	Wang et al. (2021)
	In the US, Asian journalists reported feeling stigmatized during the COVID-19 pandemic due to their ethnicity, even just from their appearance of looking Asian “… just because of his rhetoric to Asian to Chinese people and, and to the virus, and, like, people don’t know [my ethnicity], you know, like, to them, I’m a Chinese girl, you know what I mean? Um, so to them, I’m just, I’m just a yellow-skinned person that, like, maybe doesn’t fit in at the rally. So, I’ve had to worry in that sense, even, I feel like I’ve never had to worry before. Like, I feel like, now I’m, I’m more scared than I have been before when I covered certain topics.” (p. 125)	Oh and Min (2022)
	In the US, Asian Americans were seen to report more discriminatory experiences compared to Native Hawaiians and Pacific Islanders during the COVID-19 pandemic	Park et al. (2022)
	In the US, the ethnic minorities (Blacks and South Asians) reported feeling stigmatized by the general public and the community around them “I think somehow there’s always this narrative that we’re the other. COVID, obviously there’s research and statistics showing that our communities have been affected far more greater than white counterparts. And so, that’s another problem to us, if that makes sense … We have COVID, and nobody else seems to have it. And we’re the ones that are spreading it around.” (Pakistan, Female, 22, p.146) “I think there’s also a sense of stigma in the Somali community, when it comes to illness and diseases, people don’t want to be seen as weak or vulnerable. So, I think there’s also that mentality of just firming it and hoping for the best, and not wanting to be seen as suffering from COVID-19.” (Somali, Male, 27, p.146)	Vandrevala et al. (2022)
	All racial/ethnic minorities were significantly more likely than White adults to experience COVID-19 related discrimination. However, American Indian/Alaska Native and Asians were the only participants to significantly experience COVID-19 related discriminatory behaviours compared to Whites	Strassle et al. (2022)
	In the US, university students of ethnic minority such as Asians, Blacks and others were more likely to report very often experiencing bias or hatred since the COVID-19 pandemic compared to White university students, in the following order of odds ratio: Asian and others > Black > White	Shah et al. (2021)
	In Germany, participants of Asian origin reported higher COVID-19 related discrimination and attribution of discrimination to the COVID-19 pandemic. For other ethnic minorities (origin from America or former Soviet Union), only those living in high cases of COVID-19 areas reported higher COVID-19 related discrimination compared to the Germany majority	Dollman & Kogan (2021)
	In the US, Hispanic and non-Hispanic Black were significantly more likely to experience major discrimination compared to non-Hispanic Asians and non-Hispanic White participants during the COVID-19 pandemic. All ethnic minorities reported higher COVID-19 related discrimination and anticipated discrimination compared to non-Hispanic White participants	Le et al. (2022)
	In Australia, Chinese queer women on online Western dating apps felt more stigmatized and discriminated due to their ethnicity during the COVID-19 pandemic	Li and Chen (2021)
	Asian Canadian and Asian American health-care workers reported stigmatization and discrimination by patients as well as other healthcare workers during the COVID-19 pandemic for their ethnicity “She kept on screaming that I am provoking fear and that I am the reason for this confusion for society, and that I am COVID positive, since I have a mask on. …questioning … if I was COVID positive, why am I even outside. She said I am from China, and that people like me have COVID and shouldn’t be outside.” (Nurse, Asian Canadian, p.1002)	Shang et al. (2021)
	In the US, Asians were more likely than non-Asians to report experience of discrimination during COVID-19 as well as anticipate discrimination after the pandemic	Kim et al. (2022)
	In the US, a few healthcare workers reported self-stigma due to their race during the COVID-19 pandemic “I’ve felt a lot more worried about having sort of racial attacks or racial comments pointed in my direction, given some of the narrative around COVID, such as the ‘Chinese Virus’” (p. 5)	Jesuthasan et al. (2021)
	Healthcare workers (HCW) of color reported higher discrimination compared to non-Hispanic White counterparts during the pandemic. Pandemic-related discrimination theme emerged among Asians HCWs where they felt blamed for the pandemic “I have had patients reluctant to have me be their anesthesiologist because of my Asian ethnicity and afraid that I may give them COVID”	Hennein et al. (2021)
	In the US, Asian university students reported experiencing higher discrimination during the COVID-19 pandemic compared to the White students	Inman et al. (2021)
	Chinese international students perceived self-stigma from other dominant cultural groups during the pandemic in the US. The students mostly attributed the self-stigma to their ethnicity of being Chinese. Moreover, some of the students felt stigmatized or received less support by fellow Chinese nationals back in China as they were blamed for encountering stigmatizing responses by leaving China and being in the US “The only thing special is that we are all Chinese, and we were all wearing masks.” (Male, 20, Chinese in the US, p.5) “Their [friends in China] attitude to me is why you chose to be in the USA [you asked for it]” (Female, 24, Chinese in the US, p.6)	Ji and Chen (2022)
	In the US, Blacks and Latinx students recounted discriminatory behaviours towards them during the COVID-19 pandemic due to their race while the White students’ recount of negative experiences included more of day-to-day life interruptions due to the COVID-19 pandemic “if you’re a person of colour, I’m pretty sure that you’ve felt it. It’s an unspoken thing to definitely feel it.” (Black, 23, p.9)	Tan and Umamaheswar (2021)
	Chinese American adolescents who identified most as Chinese and lowest as American (separated profile) reported highest levels of being racial discriminated during the COVID-19 pandemic compared to those who participated in both Chinese and American culture (bicultural profile) as well as those who had low levels of participation in both cultures (marginalized profile). However, all three profiles did not significantly differ on reporting levels of feeling vicarious discrimination	Zong et al. (2021)
2. Religious Identity (especially the minority)	During the COVID-19 pandemic, significant differences in perceived self-stigma was reported in Canada across religions according to prevalence rate: ‘Other’ religion > Islam > Christianism > Atheism > Judaism	Miconi et al. (2021a)
	A participant in South Asia reported feeling stigmatized during the COVID-19 pandemic for being a Muslim “As I belong to a particular religion, the people near my home keep on bullying us for the coronavirus, which is not fair” (Moslem participant in South Asia, male, in his 20 s) (p.10)	Dye et al. (2020)
	In Israel, about a third of participants from cultural minority group, Ultra-orthodox Jews reported experiencing microaggressions during the COVID-19 pandemic due to their religious identity	Weiden et al. (2021)
	In Lebanon, Muslims were associated with higher self-stigma (e.g., feeling isolated by others, internalized shame of being COVID-19 positive) compared to Christians among those who have tested COVID-19 positive	Haddad et al. (2021)
3. Language as a Minority	During the COVID-19 pandemic, significant differences in self-stigma prevalence was reported among different spoken languages groups in Canada, in the following order: English-speaking cultural group > Bilingual (English & French) > French-speaking group	Miconi et al. (2021a)
4. Cultural Values Conflict with the Dominant Society	A few Chinese students reported being stigmatized due to wearing masks during the COVID-19 pandemic and attributed it to differences in cultural values and norms “American culture values human rights and freedom. The American people do not listen to their government, even if their government asks them to do something. Chinese people tend to listen.” (p.14) “…values personal freedom more than anything else…This is what Americans looks like to me…they live a very independent and individualistic life…” (pp.14–15)	Ma and Zhan (2020)
5. Countries and Regions	Participants from Africa, Asia, Northern America, and Oceania reported a higher frequency of self-stigma (experiences of bullying, harassment, and hurt) compared to those in Latin America & Europe during the COVID-19 pandemic	Dye et al. (2020)

[Insert Table 5: Cultural factors Associated with Self-Stigma related to Pandemics].

#### Ethnic Minorities, especially Chinese in Western Countries

This was the most frequently identified cultural factor (N = 23). In North America (the USA and Canada), several studies found that Asians reported the highest degree of being stigmatized or discriminated against during the COVID-19 pandemic compared to other races, especially Whites (Inman et al., 2021; Miconi et al., 2021a, 2021b; Pan et al., 2021) and other People of Color (POC), such as Blacks or Latino/a (Molock & Parchem, 2021), or Native Hawaiians and Pacific Islanders (Park et al., 2022). Generally, in the US, Asians were seen to experience higher self-stigma compared to non-Asians during the COVID-19 pandemic (Kim et al., 2022). Moreover, some Asians attributed self-stigma to their ethnicity or race. Dye et al. (2020) reported that across 173 countries Asian and Chinese participants felt stigmatized during the COVID-19 pandemic due to their ethnicity. For instance, a Southeast Asian participant in Europe stated, “I am Asian…people treated me badly and avoided me.” (Dye et al., 2020, p.10). Similar findings were echoed among Chinese or Asians in France, and the US, whereby their Asian physical appearance, as well as China being the pandemic origin, were some of the reasons for feeling stigmatized and being discriminated against during the COVID-19 pandemic (Ji & Chen, 2022; Oh & Min, 2022; Wang et al., 2021). Interestingly, Ji and Chen (2022) indicated that the Chinese international students not only felt stigmatized by the majority in the US, but also felt blamed and received less support from the Chinese back in China, as they were told it was their own choice and responsibility for leaving China and studying in the US.

Moreover, self-stigma during the pandemic was not limited to Asians per se, as other ethnic minorities also reported higher experiences of discrimination compared to the majority in the USA (e.g., Le et al., 2022, Shah et al., 2021, Strassle et al., 2022, & Tan & Umamaheswar, 2021). For instance, ethnic minorities such as Blacks and South Asians in the USA reported similar narratives where they felt that they were stigmatized by the public and the community around them such as, “I think somehow there’s always this narrative that we’re the other…We have COVID, and nobody else seems to have it. And we’re the ones that are spreading it around.” (Vandrevala et al., 2022, p.146). Interestingly, not only were particular racial labels associated with intensified self-stigma, but personal identification with the racial label was found to be associated with a higher level of self-stigma. For instance, Zong et al. (2021) found that those Chinese American adolescents who identified themselves most as “Chinese” (participation in Chinese culture) and lowest as “American” reported the highest level of racial discrimination experiences, compared to their counterparts who identified either as bicultural or had a low level of identification with both cultures. Increased self-stigma among ethnic minorities was not limited to COVID-19 pandemic, as an earlier qualitative study during SARS also found that residents of the Chinatown in New York, USA reported feeling stigmatized due to their ethnicity (Eichelberger, 2007), such as a participant stated: “I did feel more self-conscious that I was getting these looks because I was—I am Asian, Asian–American. That was hard for me.” (p. 1290). In short, the studies showed that ethnic identity, especially being the minority, was associated with higher self-stigma during a pandemic.

#### Religious identity, especially among the minorities

Three studies found that minority religious groups were stigmatized during the COVID-19 pandemic. For example, in Quebec, Canada, individuals who self-reported as “other” religion reported the highest discrimination during the COVID-19 pandemic, followed closely by Islam and then Christianity, Atheism, and Judaism (Miconi et al., 2021a). Moreover, in Dye and colleagues’ (2020) study, a Muslim participant wrote that they felt stigmatized in a South Asian country, “As I belong to a particular religion [Islam], the people near my home keep on bullying us for the coronavirus, which is not fair” (Dye et al., 2020, p.10). Weiden et al. (2021) found that a third of the participants from the cultural minority group in Israel, Ultra-orthodox Jews, perceived they experienced microaggressions due to their religious identity during the COVID-19 pandemic. Interestingly, Haddad et al. (2021) found that Muslims in Lebanon (the majority) who were diagnosed with COVID-19 were more likely to report feeling isolated by others or having higher internalized shame concerning their condition compared to Christians who were diagnosed.

#### Being a minority by spoken language

Self-stigma was not only associated with religious minorities but also with those who were a minority by spoken language. In the same study conducted in Quebec, Canada, Miconi et al. (2021a) found that those who were English-speaking were more likely to report experiences of discrimination during COVID-19 compared to those who were bilingual (English and French) or French-speaking only. As Quebec is a French dominant region, this implies that those who could not speak the French language (most likely a foreigner) faced higher self-stigma than others during the pandemic.

#### Cultural values conflict with the dominant society

A qualitative study by Ma and Zhan (2020) found that a mismatch in cultural values was associated with stigma experiences. They interviewed Chinese international students who felt stigmatized by Americans in the USA. Chinese students reported that they were being stigmatized due to the mismatch in cultural values and norms between the Chinese and mainstream American society, which resulted in differences in health behaviors and beliefs during the pandemic. For instance, an interviewee mentioned, “American culture values human rights and freedom. The American people do not listen to their government, even if their government asks them to do something. Chinese people tend to listen.” and another, “…Americans look…to me…they live a very independent and individualistic life…” (Ma & Zhan, 2020, p.14). Another participant quoted their American Professor’s judgment of them for wearing masks, “Why are you wearing masks? You are selfish. You should leave the masks to doctors” (Ma & Zhan, 2020, p.11).

#### Countries and regions

Finally, one study found that self-reported discrimination differed among countries. Countries from African, Asian, Northern American, and Oceania regions reported higher experiences of self-stigma, represented by COVID-19 related bullying/discrimination, compared to those in Latin America and Europe (Dye et al., 2020).

## Discussion

**Fig. 2:**
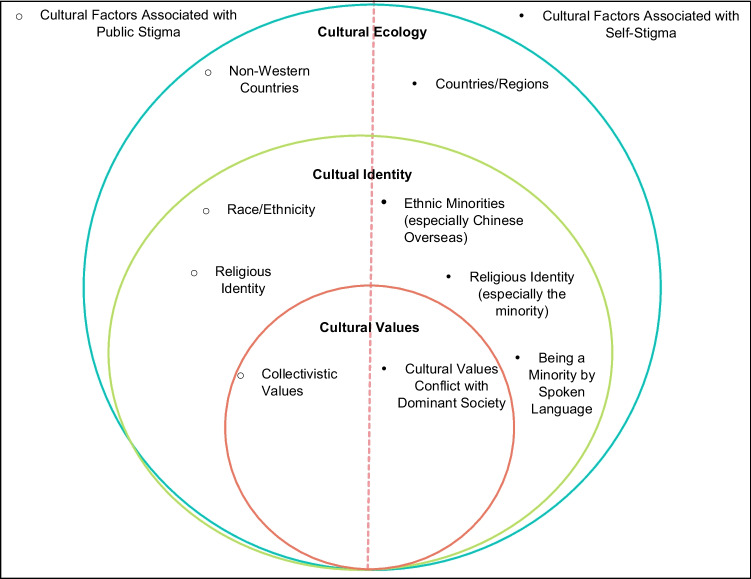
Systemic Cultural Stigma Model. Note: The Systemic Cultural Stigma Model displays factors associated with Public Stigma on the left side and factors associated with Self-Stigma on the right side

The current systematic review identified a number of cultural factors associated with public stigma and self-stigma during pandemics caused by VRI. Previous researchers have highlighted that stigma is a complex and multi-faceted phenomenon, with factors influencing stigma at different levels, including culture (e.g., Link & Phelan, 2001). Erez and Gati (2004) illustrated the intersection of a multi-level culture model from individual level to group, organizational, national to global levels. However, there has been no integrative model of how culture at different levels interact with stigma specifically. Hence, based on our findings, we attempted to construct the cultural factors we identified into a multi-level systemic model of cultural levels relevant to pandemic stigma. This consisted of culture from the micro-individual domain, meso-level shared cultural group identity, and the larger macro-society/ecology one resides in. The micro domain describes the influence of culture at the individual level, such as cultural values or beliefs. Next, at the meso-level of shared identity, we identified cultural group identities such as race/ethnicity, religion, or shared language, which influenced pandemic stigma. Lastly, the influence of culture at the regional or country level was mapped onto the macro-level domain, termed cultural ecology. We label this model tentatively as a “systemic cultural stigma model” (see Fig. 2).

Here, we discuss this cultural stigma model in relation to both public stigma and self-stigma during the pandemic from the perspective of evolutionary cognition from Strong Ties Weak Ties (STWT) cultural rationality theory (Sundararajan, 2020; Yeh et al., 2022) and social group conflicts from scapegoating theory (Allport, 1954). Then, we discuss the implications of our findings for community healthcare.

### Cultural domains and public stigma

As shown in the systemic cultural stigma model in Fig. 2, collectivistic values (individual level), ethnic/racial and religious identity (shared cultural identity), and non-western countries (cultural ecology) were associated with pandemic-related public stigma. These cultural factors could be interpreted through cultural niche-rationality theory, recently proposed by Sundararajan ‘s (2020). Cultural rationality theory posits how individuals differ in their cognition, such as reasoning about the world based on their evolutionary ecology, which influences the subsequent choice of survival behaviors (Morfett, 2018; Sundararajan, 2020). A Strong-Ties based community is described as comprised of close-knit relationships of family or kinship, with a cognitive orientation to external cues and resources (i.e., relying on strong-ties networks during hardship, shared responsibilities, and decision making), whereas weak-ties networks refer to distant relationship networks, such as acquaintances or strangers (Sundararajan, 2020; Ting & Sundararajan, 2018). With this foundation, STWT cultural rationality theory posits that an individual with a more dominant strong-ties rationality is more holistic in their cognitive style, where they perceive self in relation to those around them, aligning with the concept of related others (Sundararajan, 2020). Hence, in strong-ties dominant societies, individuals prioritize safety and wellness of family, kinship, and close friends, with fewer considerations given to those perceived as threats (regardless of in-group out-group membership), which could result in the social rejection of the latter. Values such as collectivism are privileged among strong-ties dominant societies, while individualism is privileged among weak-ties dominant societies (Sundararajan & Yeh, 2021). Research has also identified some associations between certain religions or ethnicities that historically embody kinship and bloodline as important and their members being more dominant in strong-ties rationality (Thong et al., 2022; Ting & Zhang, 2021; Yeh et al., 2022).

Public stigma, especially in the context of infectious diseases, has previously been explained through evolutionary needs, with its function to preserve group survival by distancing from those who are perceived as threats, namely the out-groups (Gilmore & Somerville, 1994; Kurzban & Leary, 2001; Saeed et al., 2020). During disasters such as pandemics, a society becomes tighter in its social norms, expecting higher conformity from its members, due to the commonly perceived threat of a contagious virus (Gelfand et al., 2021). STWT cultural rationality theory (Sundararajan, 2020) further suggests this type of reasoning is more privileged by strong-ties societies, following the evolutionary discourse, where tribal survival needs are prioritized over individual survival.

We found that the cultural factors associated with public stigma represented characteristics of strong-ties rationality at the micro-level through to strong-ties society at the macro-level. At the individual level, one study showed an association between collectivistic values and public stigma (Ahuja et al., 2020). At the group identity level, strong-ties ethnic identity was seen to have led to the social rejection of others who were more susceptible to the virus (Jiang, et al., 2021; Schmidt et al., 2020). In addition, we also found that religious groups in strong-ties societies were associated with higher public stigma toward the perceived threat, COVID-19 infected or affiliated persons (Haddad et al., 2021; Islam et al., 2021). Lastly, at the cultural ecology level, a study found higher public stigma among strong-ties countries, such as in Asia, Africa, Latin America, and the Caribbean regions compared to the weak-ties countries such as Europe, Northern America, or Oceania (Dye et al., 2020). The former regions have historically been considered as collectivistic societies in previous cross-cultural psychology literatures (Eaton & Louw, 2000; Holloway, 1990; Triandis, 2015). Past literature (e.g., Krendl & Pescosolido, 2020) on mental illness stigma has also corroborated our findings demonstrating a higher endorsement of stigma in Eastern countries (strong-ties characteristics) even towards their own in-group members due to the perceived threat to their group dynamics compared to Western countries (weak-ties characteristics). Overall, evolutionary cognition and niche could well explain why these strong-ties related cultural features are associated with public stigma, due to their association with the conservation of bloodline and in-group survival.

### Culture domains and self-stigma

As shown in our cultural stigma model, having conflicting or mismatching cultural values with the dominant society (individual level), or being a minority by ethnicity, religion, or language (shared cultural identity), or particular countries (cultural ecology) were cultural factors associated with pandemic-related self-stigma. We propose this pattern of self-stigma being perceived primarily among the minorities could be explained using scapegoating theory (e.g., Allport, 1954; Sullivan et al., 2014). According to Allport (1954), during social turbulence, “scapegoating” occurs as people displace their anger and frustration on those perceived as being minority group members, usually based on common cultural labels such as ethnicity or religion. Sullivan et al. (2014) further posited that the mechanism of displacing blame on tangible and controllable targets when situations are perceived as out of control (e.g., a crisis) brings a temporary sense of control to those of the majority. Hence, during a disastrous pandemic, this could result in minority cultural groups being the scapegoats, subject to self-stigma. We found that the majority of the articles in our review pointed to Chinese or Asian Americans as the ethnic minority group who felt most stigmatized or discriminated against during the pandemic due to their behavior of wearing a mask (Ma & Zhan, 2020), their physical appearance, or their affiliation with countries in the Asian region where the pandemic was believed to have originated (e.g., Dye et al., 2020; Eichelberger, 2007; Miconi et al., 2021a, b; Molock & Parchem, 2021; Oh & Min, 2022). Moreover, other ethnic minorities, such as Persons of Color in the US, also reported higher self-stigma, potentially as the result of scapegoating (e.g., Le et al. 2022; Tan & Umamaheswar, 2021). Other forms of cultural identity were also associated with higher self-stigma and discrimination during the pandemic, such as those who were members of a minority by religion or spoken language (Miconi et al., 2021a, b; Weiden et al., 2021). Especially in the context of religion, both dominant and non-dominant religious groups reported higher self-stigma (e.g., Haddad et al., 2021, Islam et al., 2021), suggesting religious identity is susceptible to self-stigma during a pandemic regardless of their social status. Lastly, in the larger ecology domain, one study found differences in the level of self-stigma across countries in different regions (Dye et al., 2020).

### Implications of our study for communal well-being

Our findings regarding cultural factors associated with stigma during the pandemic led to the proposed cultural stigma model introduced above. Despite the need for more research in certain cultural domains, our proposed model may guide the design of public health interventions during the recovery stage of the pandemic. We suggest national policies should consider culturally-responsive approaches to increase the effectiveness of anti-stigma campaigns in the community. For instance, in strong-ties societies, policymakers could reframe anti-stigma interventions as “communal thriving”, which promotes the idea of “togetherness”. To overcome disproportionate fear and perceived threat of marginalized groups, government agencies and Non-Governmental Organizations (NGOs) could emphasize messages and awareness campaigns on practical and tangible methods of keeping safe as a whole community regardless of group membership during the pandemic for the larger good (e.g., “We are all one family” or “We are all in the same boat”). On the other hand, since cultural minority groups in general perceived higher self-stigma, national bodies such as the health ministry through national policies or media could advocate for their pandemic control campaigns to be more culturally responsive towards less-represented groups (e.g., ethnic minorities, migrant workers, refugees). Moreover, during future pandemics, healthcare providers (e.g., public health officials, mental health professionals) could focus on increasing awareness of the scapegoating phenomena, introducing cross-cultural empathy exercises, and increasing the internal locus of control within individuals in the weak-ties society, to help reduce displaced anger and frustration toward minority groups. Overall, with more culturally responsive health campaigns, we anticipate a better quality of life at the individual level, as well as positive community-wide benefits, with fewer instances of isolation and division within the society.

### Gaps in the literature and recommendations for future studies

Our review highlighted that there is limited rigorous and empirical literature exploring the role of culture in pandemic-related stigma. While differences in cultural characteristics such as ethnocultural identity associated with public stigma or self-stigma were reported, cultural theories explaining those observed differences were absent. In addition, the majority of the reviewed articles only used self-reported cultural characteristics. There is a need for researchers to also use cultural scales to assess participants’ underlying cultural values (e.g., collectivism/individualism), rationality (i.e., STWT), thinking styles, etc. Moreover, the self-reported cultural characteristics, such as ethnicities, were compared with unequal sample sizes which, while reflective of the population in those countries, may limit the power of the analyses. As the current review led to our proposed cultural stigma model (see Fig. 2), we recommend that future studies consider assessing all three domains to gain a more comprehensive understanding of the role of culture. Specifically, more studies could involve cultural measures in both the individual and ecology domains, to examine the dynamic interaction between the different cultural domains. There were also limited studies examining public stigma, as the majority studied only self-stigma during the pandemic. Understanding both constructs of stigma is important, as the systematic review showed that the cultural factors associated with each differed. Furthermore, there were multiple stigma scales adopted in the different studies, which only tapped into certain aspects of stigma, such as fear (e.g., Wong & Sam, 2011) or discriminatory treatment (e.g., Dye et al., 2020), making comparisons across studies difficult. Some scales were only validated in one single national sample. Hence, we also recommend either exploring stigma concepts that may be specific to a culture through a bottom-up approach, or developing and validating standardized pandemic stigma scales for future research that are suitable for cross-cultural comparisons.

### Limitations of this study

As this systematic review only included journals published in English, it is possible we may have missed indigenous or international papers published in other languages. Moreover, due to the limited representation of countries (e.g., most studies were from the USA), there is a need to exercise caution in interpreting the current review, as there is a limit to the generalizability of the findings. Regarding the systemic cultural stigma model, there was only one study each that mapped onto the cultural values and ecology domain, due to the limited availability of empirical studies in these cultural domains relating to pandemic stigma. Lastly, as this study only reviewed cultural factors and their association to stigma, other possible contributing factors to stigma, such as sociodemographic variables (e.g., age, gender, SES, political affiliation) or personal variables (beliefs and motivation), have not been taken into consideration.

## Conclusion

The findings from our systematic review highlighted the interplay between culture and pandemic stigma, as well as informing implications for interventional campaigns at multicultural societies. We examined the intersectionality of three cultural domains, individual cultural values, cultural identity, and cultural ecology, and incorporated STWT cultural rationality theory and scapegoating theory, to draw on both evolutionary and sociological perspectives to explain how culture may affect pandemic stigma. We also highlighted the need for more mixed-method research in non-Western societies to look at both types of stigma. Finally, we advocate that it is essential to promote culturally-responsive stigma interventions in the community, informed by research, to increase the well-being and inclusivity of minority groups during a pandemic.

## Data Availability

All articles involved in this systematic review are available through e-databases. The data generated during and/or analyzed during the current study are available from the corresponding author on reasonable request.
